# Lack of association between SOCS3 and SOCS7 polymorphisms and psoriasis

**DOI:** 10.1002/iid3.695

**Published:** 2022-09-15

**Authors:** Merve Özçep, Nilhan Atsü, Nilgün Solak, Sevim Karakaş Çelik

**Affiliations:** ^1^ Department of Molecular Biology and Genetics Bulent Ecevit University Zonguldak Turkey; ^2^ Faculty of Health Sciences Istanbul Kent University Istanbul Turkey; ^3^ Department of Dermatology Memorial Ankara Hospital Ankara Turkey; ^4^ Department of Medical Genetics Bulent Ecevit University Zonguldak Turkey

**Keywords:** cytokine signal suppressors, polymorphism, psoriasis, rs3748726, rs4969169, SOCS3, SOCS7

## Abstract

**Background:**

Psoriasis is a common, chronic, inflammatory skin disease that involves changes taking place as a result of activation of the immune system. Suppressor of cytokine signaling proteins (SOCS) are intracellular proteins that act as endogenous inhibitors of proinflammatory pathways triggered by various cytokines. In this study, the relationship between psoriasis disease and SOCS gene polymorphisms is investigated in relation to the pathogenesis of psoriasis to clarify the psoriasis susceptibility profile.

**Methods:**

The SOCS3 rs4969169 and SOCS7 rs3748726 polymorphisms were detected using the polymerase chain reaction–restriction fragment length polymorphism (PCR‐RFLP) method. The study was approved by the Clinical Research Ethics Committee of Bulent Ecevit University and performed in accordance with the ethical standards established in the 1964 Declaration of Helsinki and later amendments. All participants were informed of the parameters of the study, and they signed consent forms before being included. Statistical analysis was performed using the SPSS 18.0 (SPSS Inc.) package program.

**Results:**

For the SOCS3 rs4969169 genotype frequency, the CC/CT genotypes represented 67%/33% in the patient group and 73%/27% in the control group. For the SOCS7 rs3748726 genotype frequency, the TT/TC/CC genotypes made up 89%/9%/1% in the patient group and 91%/8%/1% in the control group.

**Conclusion:**

The polymorphisms of SOCS3 rs4969169 and SOCS7 rs3748726 were found to have no effective role in the pathogenesis of psoriasis. This is the first study to investigate this topic, and further studies with larger, more ethnically diverse samples are encouraged.

## Graphical abstract

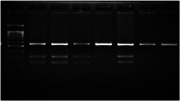

## 1 INTRODUCTION

Psoriasis is a chronic inflammatory skin disease characterized by psoriatic scales, with erythematous and sharply demarcated papules or plaques.^1^ Although the prevalence of psoriasis varies according to countries, the disease can be observed at any age. Also, ethnicity, genetics, and environmental factors can influence the onset of psoriasis. Genetic factors play an important role in its pathogenesis. Psoriasis susceptibility 1 (PSORS1), located within a segment of approximately 220 kb of the major histocompatibility complex on chromosome 6p21, is the major susceptibility locus. HLA‐Cw6 is the susceptibility allele in PSORS1; associated with early‐onset, severe disease. In individuals genetically, triggers can reveal the disease.^2^

Psoriasis is an inflammatory skin disease mediated by cells of the innate or adaptive immune systems. The immune system activated in psoriasis represents background immune amplifications that exist as essential or inducible pathways in normal human skin. These include epidermal keratinocytes, key participants in innate immunity, which can induce and replace T cells that recruit into the skin.^3^ Besides the genetic predisposition to accept psoriasis as an autoimmune disease, the biochemical pathway is similar in other autoimmune diseases such as Crohn's disease, type I diabetes and rheumatoid arthritis.^4^

Cytokine signal suppressors (SOCS) are eight families of intracellular proteins that act as endogenous inhibitors of proinflammatory pathways triggered by various cytokines. These protein families consist of SOCS1, SOCS2, SOCS3, SOCS4, SOCS5, SOCS6, SOCS7, and cytokine‐inducible Src homology‐2 protein (CIS). The SOCS3 gene functions as a negative regulator of cytokines that activate the JAK–STAT3 pathway. Expression of the SOCS3 gene is induced by a variety of cytokines, including interleukin 6 (IL6), IL10, and interferon ɣ (IFN‐ɣ^5^). SOCS3 acts not only as an anti‐inflammatory checkpoint but also as an important protective and prosurvival molecule in various cell types. Furthermore, SOCS3 suppresses apoptosis in cancer cells, as demonstrated in renal carcinoma cells undergoing IFN‐α induced apoptosis.^6^

SOCS3 expression levels have been found to be much lower in T cells from patients with psoriasis compared with T cells from healthy donors. Keratinocyte‐specific deletion of SOCS3 was found to cause psoriasis‐like skin inflammation with keratinocyte, immunoglobulin E (IgE) hyperproduction, and antimicrobial peptide expression in mice.^7^ SOCS3 is a negative regulator of the IL‐6/STAT3 signaling pathway‐related with skin homeostasis. Moreover, it has been shown that, by overexpressing SOCS3 in keratinocytes, SOCS3 aggravates wound inflammation.^8^ The deregulated IL‐6/STAT3/SOCS3 axis promoted keratinocyte proliferation, leading to the development of a severe clinical phenotype that resembled psoriasis in these transgenic mice.^7^ Therefore, SOCS3 can be seen to affect and protect against the harmful effects of prolonged proinflammatory signaling in psoriasis.

SOCS7 has been identified as having a potential tumor suppressor role. SOCS7 expression decreases with increasing tumor grade. A statistically significant relationship has been found between SOCS7 expression and cancer; in this study, the results showed that SOCS7 expression is associated with determining early tumor stage and with better prognosis and clinical outcome in breast cancer.^9^ Significantly, demonstrated that the lack of SOCS7 results in an inflammatory skin disease and that SOCS7 has a regulatory role in the production of proinflammatory cytokines by mast cells. SOCS7 inhibits IFN‐γ and IL6 signaling pathways. Therefore, the increase in SOCS7 transcript level may inhibit signaling initiated by proinflammatory cytokines.^10^

There are numerous studies investigating the relevance of SOCS in the analysis of human and animal disease models. SOCS3 activity is controlled by IL‐6, so change in serum levels of IL‐6 can affect SOCS3 expression and activity. SOCS3 is known to cause an allergic response through its Th2 activity.^11^

Single‐nucleotide polymorphisms (SNPs) may be present in other gene regions, such as 5′‐ or 3′‐untranslated regions (UTRs), introns, or promoters, as well as in the exonic region. Genetic variations in the 3′‐UTR can modify gene expression by miRNA binding, protein‐mRNA interactions, gene expression disruption, and polyadenylation; thus, SNPs in 3′‐UTRs are intriguing for researchers. Recent research has shown that SNPs in 3′‐UTRs can affect the functions of miRNAs by changing the thermodynamic properties of the hybridization site or the secondary structure of 3′‐UTR, as well as by lowering the binding yield, exchanging miRNA recognition elements (MREs), and possibly creating new binding sites or enhancing binding efficiency between the target site and miRNA.^12^, ^13^ It has been stated in several studies that 3′‐UTR‐located SNPs are useful tools for the development of medicine strategies, the assessment of disease susceptibility, and monitoring of patients' clinical symptoms.^14^

In our study, we identified 3′‐UTR SNPs altering the miRNA binding efficiency in the SOCS3 and SOCS7 genes using bioinformatics tools, and we aimed to investigate the roles of specific SNPs as follows: SOCS3 rs4969169 (C/T) and SOCS7 rs3748726 (T/C) polymorphisms in the pathogenesis of psoriasis to clarify the psoriasis susceptibility profile.

## 2 METHODS

### 2.1 Study subjects

In this study, 100 patients who were diagnosed with psoriasis among the patients who applied to the Dermatology Department of Ankara Yuksek Ihtisas University Faculty of Medicine, Private Koru Hospital, and 100 healthy individuals who were admitted as routine controls without any chronic, inflammatory, or infectious disease were included.

Psoriasis vulgaris is a clinical diagnosis and based on inspection. Typical erythematous, scaly, infiltrating plaques on the knees, elbows, and scalp are observed. The involvement of the nails, face, palmoplantar regions, and genitals may also occur. With the examination of these regions, the patients are clinically diagnosed with psoriasis. Rarely, a skin biopsy is needed. Psoriasis Area and Severity Index (PASI) score (41) was used to evaluate disease severity and extent, and patients were divided into three clinical groups; which were PASI < 3 classified as mild, 3 ≤ PASI < 10 were classified as moderate and PASI ≥ 10 were classified as a severe clinical group. Clinically, plaque psoriasis was classified in the typical group, while the others (pustular, erythrodermic, guttate, palmoplantar, plaque pustular, plaque arthropathic, guttate arthropathic, plaque guttate, and plaque palmoplantar) were classified in the atypical group. The clinical and demographic information of the study group participants is shown in Table 1. The study was approved by the Clinical Research Ethics Committee of Bulent Ecevit University and performed in accordance with the ethical standards established in the 1964 Declaration of Helsinki and later amendments. All participants were informed about the parameters of the study and signed consent forms before being included.

**Table 1:** Primer sequences and enzymes, cutting sequence and conditions utilised in the study of gene polymorphisms

**Gene**	**SNP ID**	Restriction enzymes	Cutting sequencing	Reaction condition
SOCS3	rs4969169	KpnI	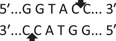	37°C
				16 h
	C/T			
SOCS7	rs3748726	NcoI	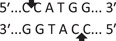	37°C
	T/C			16 h
	*Primer sequences*			
SOCS3	F: 5′ GGG ACA GGG AGC ATT TAA GG 3′			
	R: 5′ GGG AAT CTT CAA ACT TTC CAA CGG 3′			
SOCS7	F: 5′ GAG TCA TGA AGA CCC AAC AGC 3′			
	R: 5′ TCG TGT TTC CCA TCA CCT GG 3′			

Abbreviation: SNP, single‐nucleotide polymorphism.

### 2.2 In silico analysis

Using the NCBI‐dbSNP (https://www.ncbi.nlm.nih.gov/snp/) database, we determined the polymorphisms for SOCS3 and SOCS7 genes according to a minor allele frequency (MAF) > 0.15. The online database MirSNP (http://bioinfo.bjmu.edu.cn/mirsnp/search/) contains SNPs in miRNA–mRNA binding sites. Using this bioinformatics tool, polymorphisms causing changes in the miRNA binding region in the 3′‐UTR of SOCS3 and SOCS7 genes were determined.

### 2.3 Genotype analysis

DNA isolation was performed according to the manufacturer's protocol using PureLink® Genomic DNA Mini Kit (Invitrogen by Life Technologies). Polymerase chain reaction (PCR) restriction fragment length polymorphism (RFLP)‐based analysis was used to genotype SOCS3 rs4969169 (C/T) and SOCS7 rs3748726 (T/C) polymorphisms using PCR primers (Table 1).

Appropriate primers were designed for amplification of regions containing SOCS3 rs4969169 (C/T) and SOCS7 rs3748726 (T/C) polymorphisms. In the primer design, NCBI's primer design tool Primer‐BLAST (https://www.ncbi.nlm.nih.gov/tools/primer-blast/) and PerlPrimer application were used. The designed primers were checked with the online calculator OligoCalc (Oligonucleotide Properties Calculator‐http://biotools.nubic.northwestern.edu/OligoCalc.html). PCR was performed in a total reaction volume of 25 μl for each sample. The following ingredients were added to the reaction tube: 1 mM 1× Taq Buffer, 1.5 mM MgCl_2_, 0.2 mM dNTP mix, 30 pmol primers, 25–50 ng DNA, and 1 U Taq polymerase enzyme.

Amplification conditions for the SOCS3 rs4969169 (C/T) polymorphism were initial denaturation at 95°C for 3 min, followed by 35 amplification cycles of 60 s at 95°C, 90 s at 58°C, and 60 s at 72°C, with a final 7 min extension step at 72°C. The 547 base pair (bp) PCR products were digested overnight with the KpnI restriction enzyme (Table 1).

**Figure 1:**
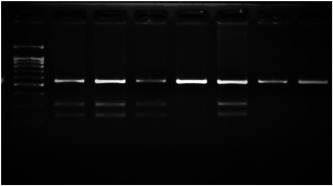
Results of SOCS3 rs4969169 gene polymorphism after enzyme digestion. DNA Ladder (marker) was loaded into first well. Each subsequent well contains restriction products belonging to a different individual. CT genotype is observable in wells 1, 2, 3, 5 identified with 547, +306, +236 bp. Whereas CC genotype is detectable in wells 4, 6, 7 with as band number 547 bp. There is no TT genotype in the groups. bp, base pairs.

**Figure 2:**
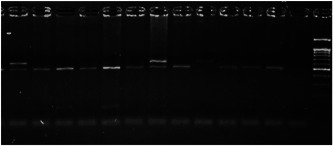
Results of SOCS7 rs3748726 gene polymorphism after enzyme digestion. DNA Ladder (marker) was loaded into the last well. Previous wells contain restriction products belonging to different individuals. CC genotype in well 9 seen as 591 bp. In wells 2, 3, 4, 5, 6, 8, 10, 11, 12 490 + 101 bp has been identified. TT genotype is evident in wells 1, 7 and 591 + 490 + 101 bp TC genotype is observed in the other wells. bp, base pairs.

Amplification conditions for the SOCS7 rs3748726 (T/C) polymorphism were initial denaturation at 95°C for 3 min, then 35 amplification cycles of 60 s at 95°C, 90 s at 57°C, and 60 s at 72°C, with a final 7 min extension step at 72°C. The 591 bp PCR products were digested overnight with the NcoI restriction enzyme (Table 1). The PCR products digested with the appropriate restriction enzyme, were analyzed by electrophoresis in a 3% agarose gel according to band sizes (Figures 1 and 2).

### 2.4 Statistical analysis

The *χ* ^2^ test was used to compare the genotype frequency of polymorphisms between psoriasis patients and healthy controls in the case‐control study. The relationship between polymorphisms and psoriasis was modeled using logistic regression analysis. To compare the psoriasis risk between genotypes, the odds ratio (OR) value and 95% confidence interval (CI) were calculated. A *p* < .05 was considered statistically significant. Statistical analysis was performed using the SPSS 18.0 (SPSS Inc.) package program.

## 3 RESULTS

A total of 200 persons were studied as follows: psoriasis patients: 55 women, 45 men; healthy control group: 58 women, 42 men. There was no statistically significant difference between the two groups in terms of gender (Table 2; *p* = .499). The subjects' age ranged between 15 and 80 years. The age of the patient group was 20–80 years, and the mean age was 40.80 ± 13.12 years. The age of the control group was 15–69 years, and the mean age was 38.97 ± 11.83 years. There was no statistically significant difference between the two groups in terms of age (Table 2; *p* = .324).

**Table 2:** Distribution of psoriasis patients and healthy control groups by gender and age

	**Healthy controls**, *n* (%)	Psoriasis patients, *n* **(%)**	*p* Value
Female	58 (58)	55 (55)	.499
Male	42 (42)	45 (45)	
Age	38.97 ± 11.83	40.80 ± 13.12	.324

Using the MirSNP database, miRNAs binding to the SOCS3 rs4969169 and SOCS7 rs3748726 gene polymorphisms were determined (Table 3; Figures 1 and 2). Allele and genotype distributions in the psoriasis patients and control groups for SOCS3 rs4969169 (C/T) and SOCS7 rs3748726 (T/C) polymorphisms are shown in Table 4. When psoriasis patients and the healthy control group were examined in terms of SOCS3 rs4969169 (C/T) gene polymorphism, no statistically significant difference was found between the two groups (*p* = .441; OR = 1.332; CI = 0.726–2.444). In addition, no statistically significant difference was found when the control and patient groups were compared in terms of allele frequencies (*p* = .484; OR = 1.266; CI = 0.730–2.197).

**Table 3:** Effects of SOCS gene polymorphism on miRNAs

GeneSNP	miRNA	Effect	Allele	Score	Energy	Binding
*(a) SOCS3 rs4969169 polymorphism*						
SOCS3 rs4969169	hsa‐miR‐299‐3p	Enhance	C	142.00	−16.77	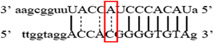
			T	146.00	−6.87	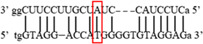
	hsa‐miR‐3150b‐3p	Enhance	C	143.00	−13.98	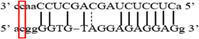
			T	145.00	−14.20	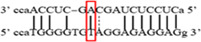
	hsa‐miR‐3151	Break	C	164.00	−27.41	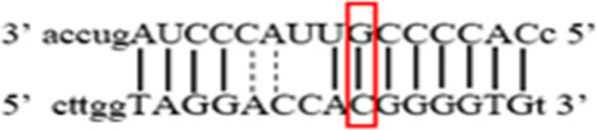
			T	‐	‐	‐
	hsa‐miR‐3605‐5p	Decrease	C	152.00	−19.36	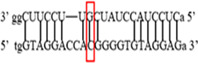
			T	150.00	−16.97	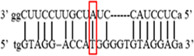
	hsa‐miR‐4733‐3p	Break	C	140.00	−14.91	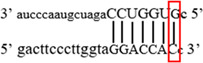
			T	‐	‐	‐
	hsa‐miR‐593‐5p	Break	C	151.00	−22.86	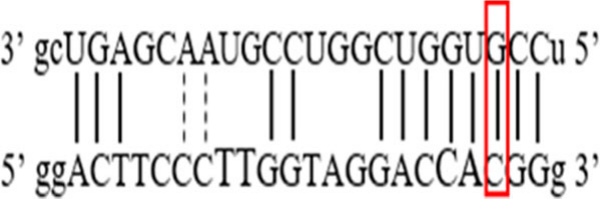
			T	‐	‐	‐
(b) *SOCS7 rs3748726 polymorphism*						
SOCS7 rs3748726	hsa‐miR‐3187‐3p	Break	T	164.00	−29.21	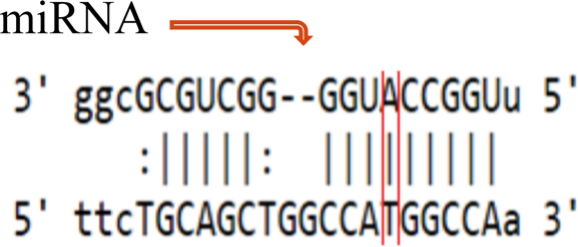
			C	‐	‐	‐
(c) *SOCS7 rs3748726 polymorphism*						
SOCS7 rs3748726	hsa‐miR‐132‐5p	Create	T	‐	‐	‐
			C	141.00	−18.28	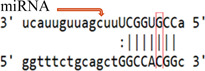
	hsa‐miR‐1204	Create	T	‐	‐	‐
			C	146.00	−18.22	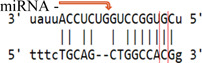

Abbreviations: miRNA, microRNA; SNP, single‐nucleotide polymorphisms.

**Table 4:** Genotype and allele frequencies of SOCS gene polymorphisms in psoriasis patients and healthy controls

SNP	Genotype	Healthy controls (*n* = 100)	Psoriasis patients (*n* = 100)	*p* Value	OR (95% CI)
SOCS3 rs4969169	CC	73 (73%)	67 (67)	.441	1.332 (0.726–2.444)
	CT	27 (27%)	33 (33%)		
SOCS7 rs3748726	TT	91 (91%)	89 (89%)	.810	Reference
	TC	8 (8%)	9 (9%)		0.489 (0.088–2.703)
	CC	1 (1%)	2 (2%)		0.563 (0.091– 3.493)
rs4969169	C	(86) 86%	(83) 83%	.484	Reference
	T	(14) 14%	(17) 17%		1.266 (0.730–2.197)
rs3748726	T	(95) 95%	(93) 93%	.668	Reference
	C	(5) 5%	(7) 7%		1.321 (0.565–3.087)

Abbreviations: CI, confidence interval; OR, odds ratio; SNP, single‐nucleotide polymorphisms.

When psoriasis patients and the healthy control group were examined in terms of SOCS7 rs3748726 (T/C) gene polymorphism, no statistically significant difference was found between the two groups (*p* = .810; OR = 0.489; CI = 0.088–2.703). In addition, no statistically significant difference was found when the control and patient groups were compared in terms of allele frequencies (*p* = .668; OR = 1.321; CI = 0.565–3.087) (Table 5). Also genotype distributions of SOCS3 and SOCS7 gene polymorphisms was compared between the typical patients group and the atypical patients group and according to Psoriasis Area and Severity Index (PASI), however there is no statistically significant relationship was found (Table 6). In our study, no significant relationship was found between SOCS3 rs4969169 (C/T) and SOCS7 rs3748726 (T/C) polymorphisms in terms of haplotype.

**Table 5:** Haplotype distributions of SOCS3 rs4969169 and SOCS7 rs3748726 gene polymorphisms in psoriasis patients and healthy controls

Haplotype					
SOCS3 rs4969169	SOCS7 rs3748726	Healthy controls (*n* = 100)	Psoriasis patients (*n* = 100)	*p* Value	OR (95% CI)
CT		(4) 4%	(5) 5%	.714	Reference
CC		(1) 1%	(1) 1%	.773	0.727 (0.084–6.314)
TC		(82) 82%	(78) 78%	.433	0.688 (0.269–1.754)
TT		(12) 12%	(15) 15%	.847	0.902 (0.315–2.583)

**Table 6:** Comparison of genotype distributions of SOCS3 and SOCS7 gene polymorphisms between the typical patients group and the atypical patients group and comparison of groups according to Psoriasis Area Severity Index (PASI)

		Psoriasis Area Severity Index (PASI)				
SNP	Genotype	Healthy controls, *n* (%)	Mild PASI < 3, *n* (%)	Moderate 3 ≤ PASI < 10, *n* (%)	Severe PASI ≥ 10, *n* (%)	*p* Value
rs3748726	TT	91 (91.0)	51 (91.1)	32 (94.1)	6 (60.0)	.057
	TC	8 (8.0)	5 (8.9)	2 (5.9)	2 (20.0)	
	CC	1 (1.0)	0 (0)	0 (0)	2 (20.0)	
rs4969169	CC	73 (73.0)	39 (69.6)	19 (55.9)	9 (90.0)	.123
	CT	27 (27.0)	17 (30.4)	15 (44.1)	1 (10.0)	

SNP	Genotype	Healthy controls, *n* (%)	Typical psoriasis patients, *n* (%)	Atypical psoriasis patients, *n* (%)	*p* Value
rs3748726	TT	91 (91.0)	75 (88.2)	14 (93.3)	.874
	TC	8 (8.0)	8 (9.4)	1 (6.7)	
	CC	1 (1.0)	2 (2.4)	0 (0)	
rs4969169	CC	73 (73.0)	57 (67.1)	10 (66.7)	.651
	CT	27 (27.0)	28 (32.9)	5 (33.3)	

## 4 DISCUSSION

Psoriasis is a disease, that is, common in the population and progresses in a chronic form, with periods of attack and remission. Its incidence in the general population is accepted to be 1.5%–2%. Although the etiology of psoriasis is not clearly known, there is wide acceptance of the idea that it is multifactorial.^15^

Cytokine signaling depends on the activation of intracellular molecules, including JAKs and STATs. Targeting analyses of SOCS molecules have revealed their essential role in the negative regulation of various cytokines in vivo. Moreover, consistent with the pathological effects of cytokines in diseases, recent evidence suggests that SOCS molecules play a role in autoimmunity, allergies, and cancers. The SOCS family of proteins are cytokine‐inducible inhibitors of the JAK–STAT signaling pathways.^16^

Sonkoly et al. reported that SOCS3 expression decreased in psoriatic epidermal keratinocytes. In addition, IFN‐α‐mediated induction of SOCS3 expression in psoriatic T cells was found to be significantly lower compared to non‐psoriatic T cells. In this case, it was associated with IFN‐α‐induced JAK and STAT activation.^17^ Equally, hundreds of miRs are expressed in keratinocytes and immune cells and play essential roles in regulating their development and function. Expression profiling studies have revealed that many miRs are dysregulated in keratinocytes and lymphocytes from psoriasis patients.^18^

In our study, the score value is the miRNA–mRNA binding score predicted by the miRanda software. The higher this value, the more stable the binding. The value expressed as energy is the free energy of the miRNA–mRNA duplex. Low‐duplex free energy indicates that the miRNA–mRNA link is stable. For the SOCS3 rs4969169 (C/T) gene polymorphism, when the T allele is replaced with the C allele, the miRNA–mRNA binding of the hsa‐miR‐299‐3p and hsa‐miR‐3150b‐3p miRNAs increases, but the miRNA–mRNA binding activity of the hsa‐miR‐3151, hsa‐miR‐4733‐3p, and hsa‐miR‐593‐5p miRNAs disappears. In addition, for hsa‐miR‐3605‐5p, miRNA–mRNA binding decreases. For the SOCS7 rs3748726 (T/C) polymorphism, when the C allele is replaced with the T allele, the miRNA–mRNA binding activity for hsa‐miR‐3187‐3p disappears. For hsa‐miR‐132‐5p and hsa‐miR‐1204, miRNA–mRNA binding forms a new junction. However, when the SOCS3 rs4969169 gene polymorphism genotype distribution and allele frequencies were analyzed, no statistically significant difference was found between the patient and control groups. Similarly, Yan et al.^19^ found no statistically significant relationship between the SOCS3 rs4969169 gene polymorphism and Graves' disease. Furthermore, Ekelund et al.^20^ found no significant relationship between SOCS3 3′‐UTR rs4969168 gene polymorphism and atopic dermatitis (AD). However, a statistically significant relationship was identified between the rs12952093 and rs4969170 polymorphism haplotypes in the SOCS3 promoter region. The A‐A haplotypes of the rs12952093 and rs4969170 polymorphisms in the SOCS3 promoter region were positively associated with AD, and the C‐G haplotypes were negatively associated with AD.

Persico et al.^21^ investigated the relationship between SOCS3 rs12952093 and rs4969170 rs4969168 gene polymorphisms and hepatitis C virus infection. A significant relationship was recognized between SOCS3 basal expression levels and hepatitis C. In particular, the SOCS3 rs4969170 AA genotype has been strongly associated with failure of antiviral therapy. SOCS3 mRNA and protein levels were significantly higher in the AA genotype carriers. Basal levels of SOCS3 and SOCS3 polymorphisms, which inhibit JAK–STAT pathways induced by IFN‐α, have been shown to affect the outcome of antiviral therapy.

Hoan et al.^22^ determined that the SOCS3 gene promoter rs111033850 T/C polymorphism contributed to the reduction of the risk of hepatitis B virus (HBV) infection, while the rs12953258 C/A polymorphism caused increased susceptibility to HBV infection. In addition, it has been shown that SOCS3 mRNA expression is significantly higher in HBV‐associated hepatocellular carcinoma (HCC) tissues. This shows that SOCS3 gene polymorphisms and methylation play an important role in the regulation of SOCS3 expression, affecting the progression of HBV‐associated liver diseases. SOCS3 polymorphisms inhibit the JAK/STAT signal by modulating SOCS3 protein expression. In this case, this supports the notion that SOCS3 affects the progression of liver disease.

When the SOCS7 rs3748726 gene polymorphism genotype distribution and allele frequencies were analyzed, no significant difference was found between the patient and control groups. Zhang et al.^23^ investigated sepsis sensitivity after blunt trauma injuries and identified a significant relationship between the SOCS7 rs3748726 T/C gene polymorphism and sepsis sensitivity.

Few studies in the literature have examined the interactions between SOCS3 and SOCS7 gene polymorphisms and immune diseases, and more studies are needed to elucidate the effect of SOCS on psoriasis because of the complex pathogenesis of this disease. Our study is the first to investigate the possible effects of rs4969169 and rs3748726 in psoriasis. However, a limitation of our study that needs to be acknowledged is the small sample size of our population. Published data on the prevalence of psoriasis in different countries varies between 0.1% and 11.8%, and the prevalence in developed countries is between 1.5% and 5%.^24^ Since the prevalence of the disease varies according to geographic region, our study contributes to the growing body of research. Further, we encourage our international colleagues to use our research findings as we collectively seek a greater understanding of the impact SOCS genes have on psoriasis.

## 5 CONCLUSION

This is the initial study investigating the possible effects of rs4969169 and rs3748726 in psoriasis. The study determined that rs4969169 and rs3748726 polymorphisms do not play an active role in psoriasis pathogenesis. Future studies that include a larger, more ethnically diverse sample to consider this issue are encouraged.

## CONFLICT OF INTEREST

The authors declare no conflict of interest.

## Data Availability

Data are available upon request to corresponding author.
